# A method for estimating maternal and newborn lives saved from health-related investments funded by the UK government Department for International Development using the Lives Saved Tool

**DOI:** 10.1186/s12889-017-4748-z

**Published:** 2017-11-07

**Authors:** Ingrid K. Friberg, Angela Baschieri, Jo Abbotts

**Affiliations:** 1 Division of Health Services, Norwegian Institute of Public Health, Oslo, Norway; 2 London School of Hygiene and Tropical Medicine, Faculty of Epidemiology and Population Health, London, UK; 3 Department for International Development, East Kilbride, UK

**Keywords:** Modelling, Lives Saved Tool, Maternal mortality, Newborn mortality, Development assistance, Department for International Development (DFID), Reproductive, Maternal, Newborn

## Abstract

**Background:**

In 2010, the UK Government Department for International Development (DFID) committed through its 'Framework for results for reproductive, maternal and newborn health (RMNH)' to save 50,000 maternal lives and 250,000 newborn lives by 2015. They also committed to monitoring the performance of this portfolio of investments to demonstrate transparency and accountability. Methods currently available to directly measure lives saved are cost-, time-, and labour-intensive. The gold standard for calculating the total number of lives saved would require measuring mortality with large scale population based surveys or annual vital events surveillance. Neither is currently available in all low- and middle-income countries. Estimating the independent effect of DFID support relative to all other effects on health would also be challenging.

**Methods:**

The Lives Saved Tool (LiST) is an evidence based software for modelling the effect of changes in health intervention coverage on reproductive, maternal, newborn and child mortality. A multi-country LiST-based analysis protocol was developed to retrospectively assess the total annual number of maternal and newborn lives saved from DFID aid programming in low- and middle-income countries.

**Results:**

Annual LiST analyses using the latest program data from DFID country offices were conducted between 2013 and 2016, estimating the annual number of maternal and neonatal lives saved across 2010–2015. For each country, independent project results were aggregated into health intervention coverage estimates, with and in the absence of DFID funding. More than 80% of reported projects were suitable for inclusion in the analysis, with 151 projects analysed in the 2016 analysis. Between 2010 and 2014, it is estimated that DFID contributed to saving the lives of 15,000 women in pregnancy and childbirth with health programming and 88,000 with family planning programming. It is estimated that DFID health programming contributed to saving 187,000 newborn lives.

**Conclusions:**

It is feasible to estimate the overall contribution and impact of DFID’s investment in RMNH from currently available information on interventions and coverage from individual country offices. This utilization of LiST, with estimated population coverage based on DFID program inputs, can be applied to similar types of datasets to quantify programme impact. The global data were used to estimate DFID’s progress against the Framework for results targets to inform future programming. The identified limitations can also be considered to inform future monitoring and evaluation program design and implementation within DFID.

**Electronic supplementary material:**

The online version of this article (10.1186/s12889-017-4748-z) contains supplementary material, which is available to authorized users.

## Background

The UK Government is committed to reducing poverty in low- and middle-income countries and has supported global actions toward achievement of the UN Millennium Development Goals (MDGs), including MDG4 (reducing child mortality) and MDG5 (improving maternal health). To support attainment of the MDGs, the Department for International Development (DFID) announced their ‘Framework for results for reproductive, maternal and newborn health (RMNH)’ in 2010, which highlighted two strategic objectives: preventing unintended pregnancies and ensuring safe pregnancies and childbirth [1]. This framework included five main goals for reproductive, maternal and newborn health and specific commitments to save the lives of at least 50,000 women during pregnancy and childbirth and 250,000 newborn babies by 2015 [1]. The framework also included a commitment to monitor the performance of this portfolio of investments.

Currently, cost-effective methods are not available to directly and comprehensively measure maternal and newborn lives saved as a result of the varied types of health programs and funding streams supported by the UK Government. The ideal method would be direct measurement of mortality through annual vital registration data or population-based surveys conducted immediately before and after implementation of each project. However, measuring maternal and neonatal mortality with adequate precision requires costly and time consuming large-scale surveys and the timing of most donor programming does not easily coincide with national survey schedules. An additional challenge would be to estimate the impact attributable to DFID funded programmes versus impacts from other donors, government funds or non-health sector activities. Given the limitations and feasibility issues associated with direct measurement of mortality, modelling has the potential to estimate the maternal and newborn lives saved from the DFID funded programmes [2] and is an increasingly viable option.

A review of alternative modelling approaches conducted by DFID identified the Lives Saved Tool (LiST) [3] as their preferred modelling approach. It simultaneously considers the timing and scale of multiple interventions as well as the best scientific information about the effectiveness of each maternal and newborn health intervention. In addition, LiST is a free, publicly available software which combines the best scientific information about effectiveness of interventions for maternal, foetal, neonatal and child health with country specific information about cause of death and current population coverage of interventions to inform planning and decision-making as well as support investment prioritization processes and evaluate existing programs [4]. LiST utilizes publicly available information on demography, family planning, HIV (incidence, prevention and treatment) and coverage of health interventions to estimate the number of lives saved by changes in these characteristics. It is built into the Spectrum Policy Modelling Software [3], and has explicit linkages to Spectrum’s AIDS Impact (AIM) and Family Planning (FamPlan) modules [5].

LiST has been extensively used for strategic planning and predicting potential future lives saved [6, 7]. Few studies have used LiST for programme evaluation [8–10], with even fewer reporting across multiple programming streams and countries. This methodological paper describes a multi-country LiST-based approach to providing a retrospective assessment of the total number of lives saved from DFID investment in low- and middle-income countries between 2010 and 2015.

## Methods

The LiST module produces estimates of total maternal and newborn deaths (along with child mortality and stillbirth) and calculates lives saved from changes in health intervention coverage [11]. LiST is the result of more than 10 years of work by the Child Health Epidemiology Reference Group (CHERG) for WHO and UNICEF and more recent collaborators, who have completed a series of systematic reviews with a consistent methodology [12] on the effectiveness of interventions that impact newborn and maternal (and child and foetal) mortality. This body of work became the basis for systematically combining current knowledge of effective interventions into a single analytic package. The CHERG and others published their work in the Lancet Series on Child Mortality (2003) [13, 14], Neonatal Mortality (2005 [15] and 2014 [16]), Nutrition (2008 [17] and 2013 [18]), and Stillbirths (2011) ([19, 20]). In addition, three supplements have included updates and additional data on effectiveness of interventions (International Journal of Epidemiology 2010, BMC Public Health 2011, BMC Public Health 2013). LiST projections utilize the following default information, all of which can be easily modified by the user to reflect national or subnational realities:

1. ***Demographic*** details based on country-specific demographic projections produced by the United Nations Population Division.
2. ***Cause of death*** information for neonates, children under five, mothers, and stillbirths, from country-specific WHO profiles.
3. ***Coverage*** levels for key health interventions affecting child morality, stillbirth and maternal mortality.
4. ***Health status*** indicators such as nutritional status.
5. ***Effectiveness*** estimates on cause-specific mortality for relevant health interventions based on the latest scientific evidence.

LiST is a mathematical model which assumes a linear relationship (the effectiveness) between changes in the coverage of a health intervention and cause-specific mortality. Changes in health intervention coverage are applied sequentially along the continuum of care, with preventive interventions applied prior to curative ones. Within each time and preventive/curative category, all interventions are applied simultaneously [4].

### Project and intervention selection

**Fig. 1:**
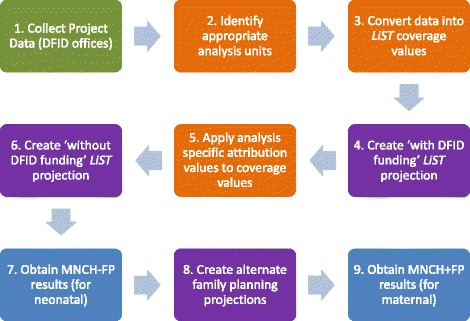
Sequence for LiST-based analysis of lives saved by DFID programming. Adapted from Friberg, Baschieri and Abbotts, 2016. Green are DFID country office tasks. Orange are processing tasks. Purple are LiST projections. Blue are results

The analysis sequence is depicted in Fig. 1 with nine primary steps. Projects conducted in any of 27 DFID focus countries were eligible for study inclusion (Fig. 1, step 1). The utilization of the Lives Saved Tool for this analysis specifically limits the scope of interventions which can be modelled. Although changes in non-health related interventions such as girls’ education, women’s empowerment, transportation, financing and socio-economic status influence health, the LiST model does not explicitly incorporate these inputs. Data on all reproductive, maternal, newborn and child health interventions were requested from the DFID country offices, although only impacts on maternal and newborn health are discussed here. Only bilateral programmes maintained and supported by the country office were included in this analysis. Multi-country programmes managed centrally by DFID headquarters and funding given directly to multilateral organizations were excluded to minimize the risk of double-counting results from programmes which overlapped geographically and in content.

### Inputs

A standard template was used to collect data from projects funded by DFID. The template was sent to each DFID country office annually to gather project-specific information on all DFID funded investments relating to health or family planning for the years 2010–2015. For each project, project-level characteristics of duration, total funding and the DFID attribution, geographical area of implementation, and the projected or actual target population sizes were documented and used to inform the specific analysis design, including the appropriate analysis units (Fig. 1; Additional file 1: Table A). The specific interventions supported by the project were identified and the forecast or projected targets and actual coverage levels, services delivered or milestones achieved for each intervention were recorded. In the annual updates, projected targets were frequently updated with evidence of activity achievements or altered targets, potentially resulting in notably different input data over time.

An estimation of the DFID attribution for each project or intervention was provided by the country offices. This was generally operationalized as the proportion of total funds dispersed relative to national government and other international donor contributions to support any programming related to RMNCH, assuming that DFID funds a direct proportion of the entire RMNCH portfolio. Some countries reported the proportion of a specific RMNCH sector, such as family planning, nutrition, etc. This analysis of specific attribution proportion was directly applied to all interventions reported by the country programs from their logframes (Fig. 1; Additional file 1: Table B).

### Analysis

**Fig. 2:**
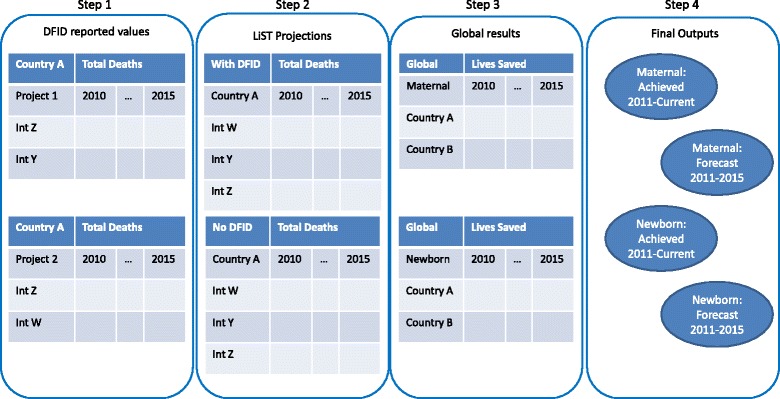
Illustration of the sequential estimation approach. Int: Intervention. The figure shows four primary sequential steps from the original raw reported values from the country offices to the final outputs. Steps 2–4 were repeated for each alternative set of mortality rates which were available

A series of LiST analyses was conducted for each country representing all of the projects which were implemented and supported in that country. Each LiST analysis consisted of two paired LiST projections or scenarios: the actual or expected situation depicting ‘with DFID support’ and a counterfactual scenario reflecting potentially no DFID funds being dispersed (‘without DFID support’). (Fig. 2; Additional file 1: Table B.) The annual difference in lives saved between the two scenarios was the primary analysis and summed across all of the individual LiST analyses to produce a national value, and then across all countries for a global estimate. These analyses incorporated all of the health and family planning activities which were supported by DFID. A secondary pair of LiST projections was created for each LiST analysis to disaggregate the impact of family planning from the impact of direct health interventions (Fig. 1, step 8).

Each individual LiST projection calculated an annual number of maternal and newborn deaths based on health intervention coverage changes relative to baseline values for the year 2010. The difference in the number of deaths calculated in each year between the paired LiST projections was the annual number of lives saved reported. These values were summed across 2011–2015 to generate the total number of lived saved (Fig. 2).

To calculate the correct coverage values for each LiST analysis and each pair of LiST projections/scenarios required disaggregating and re-aggregating each of the country’s projects by considering the geographic areas of the implementation, the type of project (broad budget support vs. targeted interventions) and the type of data available on the indicators (i.e., services delivered, commodities delivered, people reached by behaviour change messages, coverage; observed vs. forecast estimates). This process grouped geographically overlapping interventions and removed duplicated reporting of a single activity (Additional file 1, Table A). Once the correct combinations were determined, standardized assumptions were applied to convert the input data into the specific data format required for the LiST analyses, typically coverage estimates ranging from 0 to 100%.

LiST estimates the impact of interventions by linking the effectiveness of standard indicators with an effect size. The standard indicators are described within training materials and the software itself. Where DFID monitored interventions with non-LiST-standard indicators, rules were developed for deciding how to proceed. Interventions which required special consideration included breastfeeding, malaria prevention (i.e., intermittent preventive treatment of malaria in pregnancy (IPTp) or insecticide-treated bednets (ITNs)), infrastructure investment (i.e. upgrading or modifying health facilities) and family planning. Additional details are provided in the full report [21]. Two country examples of specific interventions are presented in Additional file 2.

Explanatory data analyses were performed along with sensitivity analysis using various publicly available mortality rate sources for newborns and mothers. The standard mortality estimates used were from the UN Inter-agency Group for Child Mortality Estimation (IGME) [22, 23]. Alternate values modelled were obtained from the Institute for Health Metrics and Evaluation (IHME) [24] as well as Demographic and Health Surveys (DHS), Multiple Indicator Cluster Surveys (MICS) (from 2008 to 2013) and other national sources. The completeness of coverage or service delivery inputs to the model (i.e., achieved vs. expected results) were also assessed at a national and global level.

### Outputs

Spectrum Version 5.33 was used for the fourth and most recent round of analysis out of five planned. In each round, two primary results for each of maternal and newborn lives saved were reported at the country level: 1) lives saved through the current reporting year (Achieved) and 2) expected lives saved over the entire 5-year period between 2010 and 2015 (Forecast) (Fig. 2). The achieved results included all services delivered and milestones; forecast targets were only used when no quantification of achieved results was available from any source. The annual and total number of maternal lives saved for each country and as a global estimate were reported, including maternal lives saved due to reduced fertility. An additional analysis which allowed for the disaggregation of family planning from direct health interventions was used to estimate the annual and total number of newborn lives saved, both nationally and globally. For DFID’s purposes, neonatal lives saved due to a reduction in fertility rates were not included in the primary results. The range of maternal and newborn lives saved calculated from all available alternate mortality rates was also reported.

For each country, the number of lives saved by each health intervention was reported over the duration of the project. Globally, the proportion of lives saved by each health intervention was reported. A full methodology note was published by DFID [21].

## Results

The methodology described above was applied to the DFID programming annually between 2013 and 2016, reporting on the activities supported by DFID in the years 2011–2014, with one final round planned to encompass the entire period 2011–2015. As of the fourth iteration of this analysis in 2016, 182 projects were reported on by 24 countries. Of these projects, 151 (83%) were included in the LiST analyses. Some projects were excluded because 1) no data were yet available (either no data reported or program not yet initiated) or 2) the interventions were not compatible with a LiST analysis (i.e. training programmes, or women’s empowerment programmes). (Table 1) Of the 24 countries analysed, at least 20 (83%) had some achieved data for the years 2011–2014, up from 57% (11 of 19) in the first year of the analysis. Achieved values included both survey data and project data, in approximately equal amounts. Behaviour change communications, and other distal inputs were minimal, and were centred around handwashing.

**Table 1:** Comparison of data available for and included in the analysis

	Countries Reporting (N)	Analyses (N)	Projects Analysed	Projects Reported	Percent Analysed
2010–2011	19	40	87	127	69
2010–2012	20 (19)*	47	113	146	77
2010–2013	23	53	146	178	82
2010–2014	24	54	151	182	83

*One country reported projects but the information was unable to be analysed

The analysis estimated that, between 2010 and 2014, DFID had contributed to saving the lives of 103,000 women in pregnancy and childbirth (15,000 due to maternal and child health programmes only) and 187,000 newborn babies [25], an increase from the lives of 6000 women and 16,000 newborn babies calculated for 2010–2011 [26], resulting from both the increased number of years evaluated as well as changes in input data. The greatest numbers of maternal lives saved were in Ethiopia and Bangladesh. The majority of the maternal lives saved (81%, 88,000) were from the family planning impact on reduced fertility. The greatest number of newborn lives were saved from care at birth, including skilled birth attendance and in some cases facility delivery up to the level of comprehensive emergency obstetric care. Forecast results are used by DFID internally to review progress against targets and only achieved results are published. Due to the known uncertainties around these values and expected changes in these forecast values, this paper has maintained this principle.

## Discussion

This paper describes the development, and preliminary application, of a methodology for utilizing the free publicly available software, LiST, to both retrospectively and prospectively assess the impact of DFID’s bilateral funding in low- and middle-income countries. This method has proven to be feasible, flexible, and replicable over time. The key strength of this methodology is its adaptability, as it was designed to maximize the utilization of already available programme data regardless of the underlying format (coverage or service delivery, forecast or achieved), while mitigating the risk of double-counting or over-estimating benefits across multiple countries. This application of the method is limited to projects directly influencing health intervention coverage through bilateral programming. The Lives Saved Tool proved to be adequately flexible for incorporating at least one health or family planning intervention from more than 80% of the bilateral health-related projects supported by DFID in priority countries between 2011 and 2015. This technique proved to be compatible with readily available data, without explicit linkage to LiST or the modelling requirements being specified in advance, suggesting that this method could be utilized in similar contexts external to DFID.

The precision of estimates is of considerable interest. Confidence intervals can be determined for inputs such as population size, birth rate, effectiveness estimates, and coverage. As well as confidence intervals around individual estimates, interactions among variables results in greater overall uncertainty. The standard inputs are likely to result in relatively modest error bounds. Including project data is not likely to reduce the width of confidence in interventions themselves.

Limitations to this analytical method can be primarily categorized as relating to data availability, both project-specific and globally, or modelling constraints. The data available for inputs into the model are often limited. Some reporting systems, including some DFID projects described here, only report selected ‘tracer’ interventions rather than whole programs, leading to underestimation of impact by ignoring interventions which may be more complex to monitor or implement. Using national targets as proxies for projected implementation achievements in the absence of quantified results may overestimate impact when the targets are aspirational rather than practical. Using commodities as a direct input to reflect intervention coverage assumes that all supplies reach their intended targets, which will likely overestimate impact by not considering potentially significant and uncounted losses. Programs may have been implemented subnationally, with estimates of the underlying default values required for LiST being minimally available except through additional cycles of estimation and modelling [27], thus increasing the uncertainty around the LiST outputs. More generally, for programs which reported proportional funding rather than services delivered, there is a significant reliance on the reliability and comparability of the attribution estimates provided. Over the course of the 5 years of this project, these proportions typically changed at least twice, for expected actions such as programmes ending or the introduction of new programming and for unplanned funding stoppages.

Modelling constraints are also critical limitations. Some, although relatively few, DFID projects reported behaviour change communication programming results with the number of individuals exposed to the message without documenting actual behaviour change, which may result in misestimating the lives saved, depending on the program type. This analysis was designed to assess bilateral programming only, excluding contributions to multilateral organizations, to avoid double-counting of impact while excluding some purely technical assistance programs, which do not include indicators amenable to modelling. While these constraints limit the ability to model entire programs, they do not change the validity of the interventions included.

The Lives Saved Tool has inherent model-based constraints, including uncertainty around software defaults and effectiveness estimates, with some, including mortality rates and causes of death, being modelled values themselves [23, 28]. Although LiST results have been validated against newborn mortality [9], this has not yet been done for maternal health outputs and is a critical gap. In addition, LiST is only capable of modelling interventions with a clearly defined causal pathway. Regardless of how enthusiastically donors, countries or implementers advocate for a particular intervention, without adequately strong evidence, some interventions with expected impact on mortality are excluded. LiST uses standard algorithms to estimate the quality of care during antenatal care and childbirth relative to overall coverage levels which have not yet been fully validated. All of these assumptions are likely to be inaccurate in certain environments. Additionally, the results cannot be considered a stand-alone comprehensive impact assessment for DFID programming and for future funding prioritization across the entire organization. The results are indicative of bilateral programming impacts, and exclude multilateral activities as well as cross - cutting initiatives.

Given the limitations of modelling as evaluation, the data gaps, and the unknown status of many indicators and interventions which support health, this project was considered successful as it produced evidence-based information that could be used to inform broad decision-making processes. The achieved and forecast results were used by DFID as inputs to processes which led to additional funding being made available to support commodities for newborns [29]. And within countries, achieved results were used to inform programming decisions in conjunction with other data sources.

The generalizability of this methodology to similar organizations is likely, although the comparability of those specific results would be limited by the explicit micro-decisions (i.e. about translations between indicators) that each application of this method requires. Each DFID program contributes to a complex system of government, donors, non-governmental organizations, economic trends, and secular trends, both health and non-health which together result in changes, for better or for worse, in health. In addition, there are extensive unknowns in every setting which potentially impact the actual lives saved, limiting the ability of any model to fully accomodate. Models, as always, are not measurements of truth but are simplifications for understanding reality. They cannot be used as stand-alone tools for assessing or evaluating programme value and impact. The analysis can also be used to highlight places where there is greater and lesser uncertainty about the results. For example, there is more uncertainty in estimates of the impact of health education programs (i.e. counting those exposed to a program) than commodity delivery programs (i.e. number of items procured or used) and both have more uncertainty than when information is available on coverage achieved by the desired behaviours, services, or interventions. The LiST results can be one data-driven input into discussions about the relative certainty around programming impact. Nor can the results stand alone to prioritize funding given that the inputs are not comprehensive of interventions, programs or initiatives.

The next step in evaluating this method should be the full documentation of its application to this dataset or another dataset for validation and exploration, including a complete description of data available, data quality and assumptions. Future modifications of this method, either globally or project specific, should include an adjustment factor for likely drop-offs in project effectiveness. This could include wastage factors for commodities programs or estimates of behaviour change relative to education programs. Operationalizing these estimates will be complex, but could have significant benefits in terms of precision.

## Conclusion

The use of the Lives Saved Tool to model the impacts of DFID bilateral programming on health has proven to be feasible and stable over time. In the absence of direct data collection and the challenge of diverse and heterogeneous data sources, modelling to quantify impact can be a valuable tool for programme monitoring and evaluation. The validity of any modelling to generate estimates on which decisions are made is only as good as the data and the model itself. Although modelling is not without limitations and is not intended to replace primary data collection, modelling complex and dynamic systems can be accomplished with LiST. The development and use of this LiST-based protocol to analyse results from a wide range of projects in multiple countries is unique and serves as an example of how to use modelling to support monitoring, evaluation and programme implementation on a large scale.

## Additional files

Additional file 1: Decision tree to determine how many analyses should be completed for each country and what populations they should include. (XLSX 13 kb)

Additional file 2: Two country examples. (DOCX 15 kb)
