# Supplementary material for: A method for estimating maternal and newborn lives saved from health-related investments funded by the UK government Department for International Development using the Lives Saved Tool
Source: BMC Public Health. 2017 Nov 7;17(Suppl 4):779. doi: 10.1186/s12889-017-4748-z (PMC5688469; doi:10.1186/s12889-017-4748-z)
Supplement: Supplementary file 2 — Two country examples. (DOCX 15 kb) [file 12889_2017_4748_MOESM2_ESM.docx]

# Additional File 3: Two country examples.

## Country A.

Data available: Three projects were reported. Coverage was reported for each of approximately 15 interventions, based on national surveys (one prior to 2010, two during the analysis period) and projected coverage achievements in the program plans. No services delivered were reported for any intervention. One maternal and child health project was conducted in two districts (Province A (PA) and Province B (PB)), one maternal and child health project conducted nationally and a family planning program conducted in a subnational area partially overlapping PA and PB. The proportion of the national health budget due to each project was reported.

Analysis: PV and PB were analysed separately. The national project was split into three parts, one for each of the two districts in the first project and a third for the rest of the country (National-Minus). The family planning program could not be applied to specific areas so was analysed separately (National). To estimate the attribution for PA and PB, we added the attribution from both projects working in those areas. This attribution proportion was multiplied by the total intervention coverage values reported for each district, and subtracted from the total coverage. For the National-Minus analysis, the remaining mortality was estimated by excluding the two districts. The attribution was based on only the one national program. The proportional attribution for family planning was also calculated and applied to the national analysis. Family planning was not included in the district analyses to prevent double counting with the dedicated family planning program which was larger than the other family planning activities.

Interventions example: Antenatal care – with DFID scenario. Antenatal care coverage was available from 2008, 2012 and 2014 surveys nationally and for each district. These were interpolated to get 2010, 2011, and 2013 values, while the 2015 value was a duplication of the 2014 value. For the without DFID scenario, the attribution varied from year to year, ranging from 1% to 3% of the national health budget for each district or nationally. The with DFID scenario antenatal care values were reduced by this proportion for each year.

Summary: The results from the with DFID scenario for each year were subtracted from the without DFID scenario to get the maternal and newborn lives saved. The results from all four analyses were added together to get newborn and maternal deaths averted. It is likely that this estimate is conservative as it is possible that some family planning impacts were excluded.

## Country B.

Data available: Ten projects were reported, including budget support, district programs and delivery of bednets and antimalarials. Two national surveys were available: one from before 2010 and one between 2010 and 2015. The budget support program reported a projected coverage, but no national survey could be used to cross-check achievements. The majority of national programs reported a single intervention in terms of supplies distributed or patients reached.

Analysis: The budget support program was analysed nationally. The district program was analysed separately, given the different underlying population and mortality. The remaining projects were combined into a single national analysis. Several programs reported achieved results (i.e. number of bednets delivered) and a proportion of that program which was supported by DFID (i.e. 38%).

Intervention example: Insecticide treated bednets. Two projects reported bednets in different areas and the country office indicated that they each reported separate bednets. The baseline in 2010 was calculated from the post-2010 national survey. The first project assumed 38% of the total was due to DFID. This result was added to the 100% assumed to be due to DFID from the second project for each individual year when they were delivered. It was also assumed that the nets would be useful for three years. The increased coverage was maintained for three years. Afterwards, all nets were removed from the calculation of coverage. The intervention of bednets was not part of the national budget program analysed, both due to not being reported but also to prevent double counting. If it had been reported, a different decision could have been taken based on conversation with the country office. The attribution for the without DFID scenario was maintained at the baseline for 2010 for all subsequent years.
